# Supplementary material for: Tead1a Initiates Transcriptional Priming Through the TEAD1a/YAP‐Notch1‐Spi1/Cebpα Axis to Promote Neutrophil Fate
Source: Adv Sci (Weinh). 2025 Aug 28;12(41):e05441. doi: 10.1002/advs.202505441 (PMC12591127; doi:10.1002/advs.202505441)
Supplement: Supplementary file 4 — Supplementary Dataset [file ADVS-12-e05441-s004.zip › 3-3.zip!/3-3/02-3.cluster/cluster6/Enrich/ego_BP.pdf]

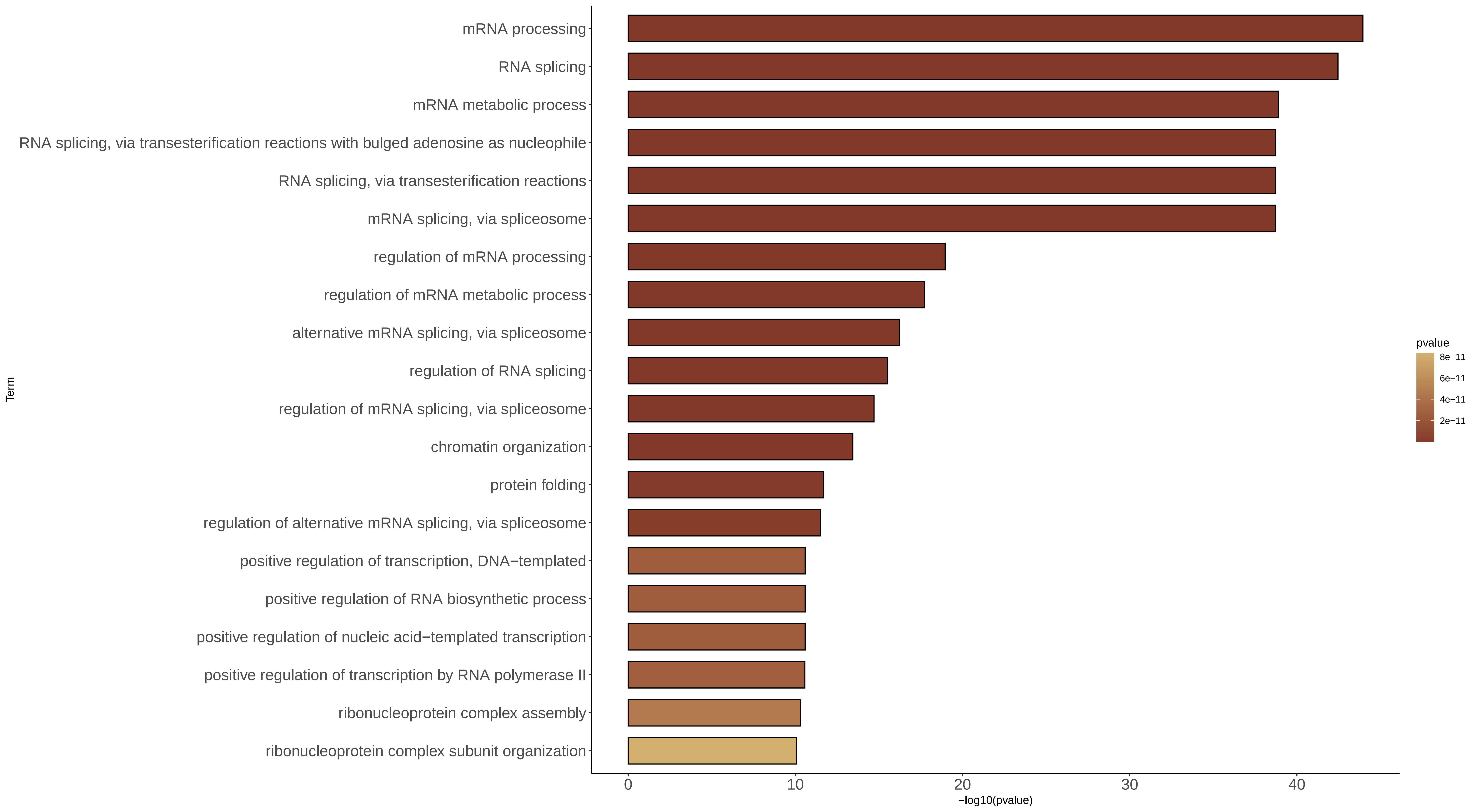

mRNA processing
RNA splicing
mRNA metabolic process
RNA splicing, via transesterification reactions with bulged adenosine as nucleophile
RNA splicing, via transesterification reactions
mRNA splicing, via spliceosome
regulation of mRNA processing
regulation of mRNA metabolic process
alternative mRNA splicing, via spliceosome
regulation of RNA splicing
regulation of mRNA splicing, via spliceosome
chromatin organization
protein folding
regulation of alternative mRNA splicing, via spliceosome
positive regulation of transcription, DNA−templated
positive regulation of RNA biosynthetic process
positive regulation of nucleic acid−templated transcription
positive regulation of transcription by RNA polymerase II
ribonucleoprotein complex assembly
ribonucleoprotein complex subunit organization
Term
0
10
20
30
40
−log10(pvalue)
pvalue
8e−11
6e−11
4e−11
2e−11
